# An *in Planta* Enrichment Route to Identify Bacterial Root Endophytes

**DOI:** 10.1111/1758-2229.70136

**Published:** 2025-06-19

**Authors:** Denise Khouri Chalouhi, Iris Bertani, Alfonso Esposito, Silvano Piazza, Cristina Bez, Vittorio Venturi

**Affiliations:** ^1^ International Centre for Genetic Engineering and Biotechnology (ICGEB) Trieste Italy; ^2^ Dipartimento di Biotecnologie Università di Verona Verona Italy; ^3^ African Genome Center University Mohammed VI Polytechnic (UM6P) Ben Guerir Morocco

**Keywords:** enrichment, nitrogen depletion, plant microbiome, rice, super endophytes

## Abstract

Microorganisms live in close association with plants, forming ecological interaction webs and providing beneficial traits such as nutrition, growth, and tolerance to biotic and abiotic stresses. Via the rhizosphere, plants recruit bacteria which colonise internal plant tissues, creating a spatial gradient between the rhizosphere and the endosphere. This study presents a high throughput *in planta* endophyte enrichment scheme designed for the identification of '*super'*‐endophytic bacteria which can serially colonise the rice root endosphere. *Oryza sativa* (rice) plants were grown in bulk soil, and endophytes were then recovered from roots. The recovered endophyte mixture was used as inoculum for the first generation of rice plantlets, which were then grown under no stress or nitrogen (N) depletion. The total endophytic community was then purified and used as a second inoculum for a new set of plants; this procedure was repeated for four generations. Enrichment patterns of root bacterial endophytes were observed, such as *Kosakonia* in the non‐stressed plants and *Ferrovibrio* in plants grown under nitrogen starvation. This enrichment method proved to be suitable for the identification of endophytes which can efficiently colonise the root endosphere.

## Graphical abstract

A serial enrichment method was developed to map root endophytes in rice across four generations. Through endophytic bacteriome analysis, this approach identified enriched bacterial genera such as *Kosakonia* and, under nitrogen‐deficient conditions, *Ferrovibrio*. This strategy has proven effective in identifying bacteria that consistently colonise rice roots.

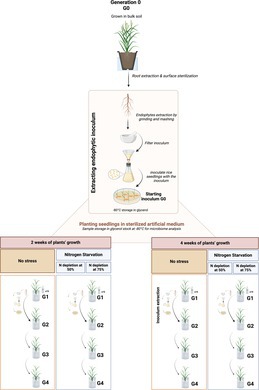

## 1 Introduction

With the current demographic growth rate, the Food and Agriculture Organisation (FAO) estimates that by 2050 we will reach 9.7 billion people. Hence, there is a need for agricultural productivity to increase by 70% in order to satisfy the food demand (Food and Agriculture Organization 2022). Moreover, sustainability of global food production is impaired by limited available land to increase agricultural production (Godfray 2010; Kopittke et al. 2019) and by the deleterious effects of climate change as well as over‐exploitation of soils which results in loss of soil fertility and biodiversity (Fowler et al. 2013). Therefore, there is a need to find alternative solutions for more environmentally friendly and sustainable fertilisation regimes (Singh and Trivedi 2017).

Microorganisms intimately associate with plants, creating a network of microbe‐microbe and microbe‐plant interactions. The majority of these microbes originate from soil, which is the richest and most diverse microbial reservoir (Hardoim 2015), and then colonise different plant‐associated compartments. One of these compartments is the root rhizosphere, the soil zone immediately surrounding and influenced by the root exudates (Zhang et al. 2023). From the rhizosphere, certain microbes move into the endosphere, the internal plant tissues (Sessitsch et al. 2002; Turner 2013). These microbes, called endophytes, possess the ability to colonise and persist within the plant's interior (Brader et al. 2017; Compant et al. 2021; Hardoim 2015). Some endophytes further migrate to other plant compartments, primarily via the vascular system, whilst others are vertically transmitted through seed inheritance (Papik et al. 2020). Endophytes interact with their hosts across a spectrum of relationships, including antagonism, commensalism, mutualism and symbiosis (Hardoim 2015). Due to their intimate and selective interaction with the plant host, bacterial endophytes are receiving attention for the diverse benefits they provide to plants. These include improved nutrient acquisition through nitrogen cycling and phosphate solubilisation, production of siderophores and phytohormones, detoxification of heavy metals and organic pollutants, and enhanced tolerance to both abiotic and biotic stresses (Compant et al. 2021; Edwards et al. 2015; Hardoim 2015; Papik et al. 2020; Turner 2013).

Endophytes are consequently being studied for the development of microbes‐based solutions to promote sustainable agricultural practises and to be used as an alternative to chemical additives (Medison 2022); a pivotal step for this application is the isolation and choice of the bacterial isolate(s). Traditional isolation and characterisation methods of endophytes from sterilised plant material are being used today without a targeted or enriched isolation procedure. Identifying beneficial endophyte, however, still presents several challenges, including (i) surface sterilisation methods to remove surface‐associated bacteria from plant material is not yet standardised, and impact the number and diversity of recovered strains (Compant et al. 2021; Dos‐Santos et al. 2017), (ii) the loss of certain endophytes during subsequent laboratory growth, as some have specific niches in the endosphere where growth is favoured or are slow‐growing bacteria with specific nutrient requirements, often outcompeted by faster‐growing strains with more dynamic metabolic capacities (Hug et al. 2016; Zhang et al. 2023), and (iii) once isolated, putative endophytes must be reinoculated and detected in the endosphere to ensure that they are true endophytes. Standardisation of isolation protocols and devising enrichment procedures will help identify true beneficial endophytes as well as in the design of multi‐strain consortia.

To our knowledge, no studies have addressed *en masse* endophyte enrichment strategy via *in planta* serial inoculations; thus, little is known about total plant endophytic transplantations as a strategy to identify/select for endophytes. In this study, using rice ( *Oryza sativa* ) as a plant model, we executed an enrichment strategy aimed at identifying endophytes, which consistently re‐colonise and persist in the endosphere. We purified rice root endophytes from rice roots grown in bulk soil and subsequently reinoculated on semi‐sterile rice roots, and then performed several subsequent serial endophytic purifications and *in planta* re‐inoculation steps. Via the identification and analysis through 16S rRNA amplicon sequencing of the bacterial endophytic community at each inoculation step, we were able to follow the dynamics of specific endophytic bacterial members.

## 2 Experimental Procedures

### 2.1 Experimental Design and Sample Collection

**FIGURE 1:**
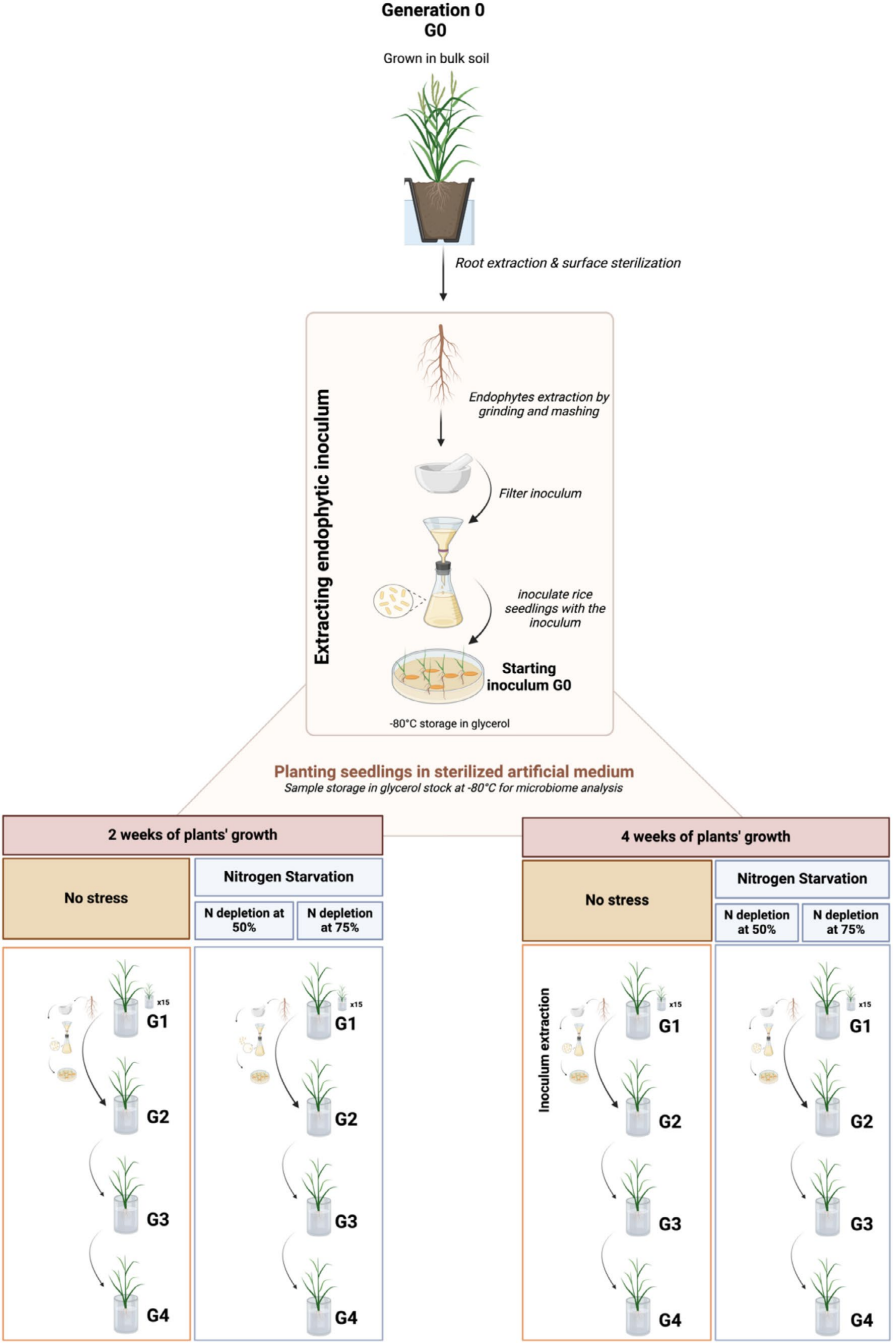
Enrichment procedure for super endophytes isolation and reinoculation.

The enrichment procedure was developed based on the rationale outlined in Figure 1, using *Oryza sativa* ssp. *japonica* Augusto cultivar as plant model. Ten seeds of this cultivar were pre‐treated by soaking in a 50% bleach solution (7% of active Cl) for 40 min and then washed with abundant sterile water. Sterile seeds were then left to germinate for 5 days on sterile humid paper in the dark in sterile boxes at 30°C. Seedlings were grown in environmental bulk soil (collected from tree woods in Padriciano, Trieste, Italy; co‐ordinates: 45.661111° N, −45.661111° W) for 42 days in the growth chamber (photoperiod 16 h of light and 8 h of darkness per day). This initial set of rice plants grown in soil was named Generation 0 (G0). From 10 rice plants (G0), *en masse* endophyte community was extracted from the roots following surface sterilisation as previously described in (Bertani et al. 2016). Briefly, rice roots were thoroughly washed with tap water and then sterilised by submerging root tissues in 75% EtOH for 2 min, then in a 50% bleach solution (7% of active Cl) for 2 min and again in 75% EtOH for 1 min. Afterwards, roots were abundantly washed with sterile water. Roots were then macerated using a mortar and pestle and adding a Phosphate Buffer Saline (PBS) solution to obtain a solution (named G0) of recovered endophytes. At this stage, aliquots of these recovered endophytes were stored in 20% glycerol stock at −80°C for 16S rRNA bacteriome analysis. This recovered mix of endophytes (G0) was used to re‐inoculate a new set of rice seedlings' roots and grown in a previously sterilised artificial medium; this new set of inoculated rice plants was named Generation 1 (G1). Re‐inoculation was performed as follows: seeds were surface sterilised, left in a 50% hypochlorite solution for 40 min then abundantly washed with sterile water. Seeds were left to germinate for 5 days in the dark on humid sterile paper and in sterile boxes at 30°C; seedlings' roots were then submerged in the bacterial suspension of G0 endophytes for 1 h (Steindler et al. 2009). Seedlings of G1 were grown in the growth chamber (photoperiod 16 h of light and 8 h of darkness per day) in semi‐solid Hoagland artificial medium (agar 0.6%) previously sterilised. Plants were grown for 2 different timespans after seedlings' inoculation, i.e., for 2 and 4 weeks after which endophytes from roots were extracted and reinoculated on a new set of seedlings' roots representing generation G2. This endophytes enrichment procedure was repeated for two more rounds, obtaining in total Generation 1, Generation 2, Generation 3 and Generation 4 (G1, G2, G3 & G4). For each generation and timespan, inoculated plants were grown in two conditions, (i) in no‐stress growing conditions (no abiotic stress applied, i.e., no modifications added to Hoagland's composition), and (ii) under nitrogen deprived conditions (nitrogen starved at 50% and 75% compared to the control). A control was also included, i.e., rice seedlings' roots inoculated with PBS only in no‐stress growing conditions. Fifteen plants per treatment were used at each enrichment step and time of growth, resulting in 390 plants in total. At each endophytic root extraction, an aliquot of the samples was stored in a 20% glycerol stock and stored at −80°C. Overall, we collected 141 endophytic samples and we performed 16S rRNA metabarcoding sequencing deriving from 4 or 5 replicas of each sample: endophytes from G1, G2, G3 and G4 of rice plants grown under 2 stress conditions tested (2 N‐concentrations × 2 timespans) and without stress (121 samples) and from the different controls, i.e., the soil in which G0 was grown, sterilised seeds, and the control group without inoculum at 2 and 4 weeks of growth (20 samples). At each treatment and enrichment step, before root sterilisation for endophytic extraction, rice roots and green parts were weighed separately. Statistical analysis was performed using ordinary one‐way ANOVA on Prism version 9.5.1.

### 2.2 DNA Extraction, Library Preparation and Sequencing

Total genomic DNA was extracted from all collected samples with the Power Soil DNA isolation kit (Qiagen) as instructed by the manufacturer. DNA quantity and quality were determined using a Nanodrop device (Thermo Scientific, Wilmington, DE). The DNA was used for the amplification of the 16S rRNA gene hypervariable regions V3 and V4 using barcoded primers. For the first amplification, barcoded individual libraries were prepared by Polymerase Chain Reaction (PCR) using long primers (Klindworth et al. 2013) having the Illumina adapter sequences that allow for the pooling of multiple samples in a single sequencing run (16S_Amplicon_PCR_Fw:TCGTCGGCAGCGTCAGATGTGTATAAGAGACAGCCTACGGGNGGCWGCAG; 16S_Amplicon_PCR_Rv: GTCTCGTGGGCTCGGAGATGTGTATAAGAGACAGGACTACHVGGGTATCTAATCC). This amplification was then cleaned using the AMPure XP bead clean‐up (A63880l; Beckman Coulter Inc., Brea, CA, USA). Then, a second amplification was made using the Nextera XT Index Kit to attach dual index and Illumina sequencing adapters and this second PCR reaction was also cleaned up using the AMPure XP beads. For these amplifications' conditions, the protocol of Illumina Inc.'s was followed (Illumina Inc., San Diego, CA, USA). Library concentration was then measured by fluorometric quantification using Qubit 2 (Invitrogen Inc., Carlsbad, CA, USA). Libraries' sequencing was performed using a 2 × 250 bp MiSeq sequencer.

### 2.3 Data Processing and Bioinformatic Analysis

For the bioinformatic analysis, fastq files were imported into Qiime2 (Bolyen et al. 2019) (version 2022.8.0) and reads were clustered into Amplicon Sequence Variants (ASVs), using the DADA2 plugin (Callahan et al. 2016). Taxonomic assignment was performed with the qiime2 classifier using SILVA database (release 138) (Quast et al. 2012). A rarefaction depth of 1000 reads was used to calculate diversity values. Afterwards, the qiime2R package (Version 0.99.6), was used to import the dataset in R (Version 4.2.2, 2022‐10‐31) (Bolyen et al. 2019) and the packages phyloseq (version 1.42.0) (McMurdie and Holmes 2013), ggplot2 (Wickham and Sievert 2016) (version 3.4.2), vegan (version v 2.6.4) (Oksanen et al. 2001) and microbiome (version 1.20.0) (Lahti 2017); were used to perform subsequent microbiome composition analyses and to draw plots (Lahti and Shahty 2012; McMurdie and Holmes 2013). To determine significantly enriched ASVs in each treatment, enrichment step, and time of growth, MaAsLin2 (package version 1.12.0) was used (Mallick et al. 2021). Permutational analysis to obtain size effects and significances for compositional differences of the bacterial community between Generation and Time was performed using adonis2 and vegan (version) (Oksanen et al. 2001).

## 3 Results

### 3.1 Rationale for the Serial Enrichments of Bacterial Endophytes

It was of interest to devise a procedure to identify and eventually isolate the best performing and colonising bacterial endophytic strains; the strategy that we implemented is depicted in Figure 1 and the methodology described in detail in the *Experimental Procedures* section. The rationale was to use the plant as a natural trap for endophytic bacteria and enrich them via serial inoculation steps. Initially, rice plants were grown in bulk soil to obtain the starting generation of root endophytes (G0), which was then used as an inoculum to re‐inoculate rice plantlets cultivated in sterile artificial media. Plants were then grown for two set times, and endophytes were purified and used to inoculate a new set of rice plantlets. This serial enrichment process was repeated across four generations (G1–G4), with the bacterial communities at each step analysed using 16S rRNA gene profiling. In parallel, a similar enrichment series was conducted with plants grown under nitrogen‐depleted conditions (50% and 75% reductions), introducing an abiotic stress factor likely to shape the composition of the endophytic community.

### 3.2 Plants Display Roots and Shoot Weight Reduction Across the Generations

**FIGURE 2:**
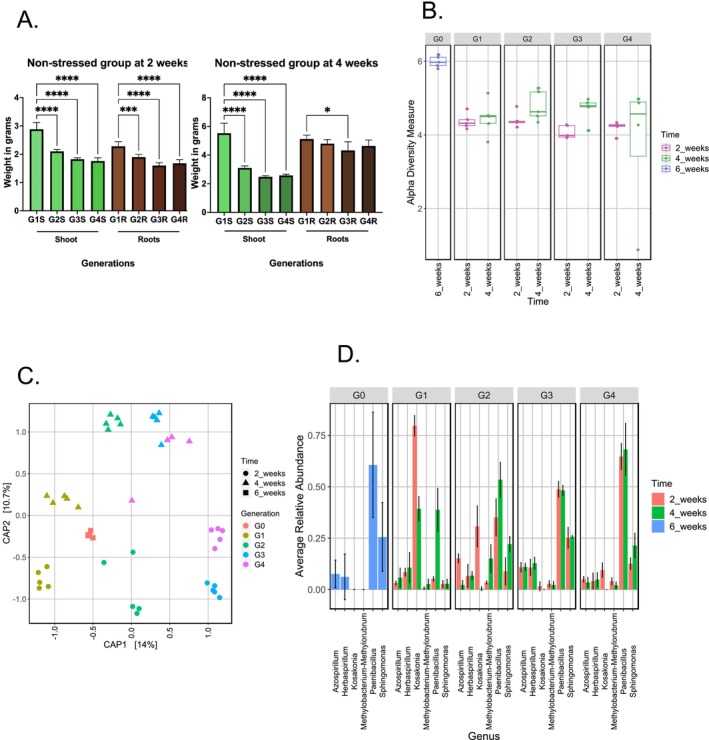
Plant growth parameters and rice root endobacteriome variations in the non‐stressed group. (a) Bar charts showing the variations of weight in grams of both roots and shoots of rice plants for the 4 generations of growth in Hoagland. The graph on the left shows data of plants grown for 2 weeks at each generation, whilst the right‐side graph shows data of plants grown for 4 weeks at each generation. Statistical analysis was performed using ordinary one‐way ANOVA on Prism. Number of * indicates degree of significance. (**** indicate *p*‐value < 0.0001; whilst * indicates *p*‐value < 0.05). (b) Alpha diversity measure using Shannon throughout the generations at both 2 and 4 weeks of growth at each generation. Colours of the box plots indicate a different time of growth. Differences are compared with the Kruskal‐Wallis test and pairwise post hoc comparisons were calculated with the Wilcoxon post hoc test. The *p*‐values corrections were made using Bonferroni correction. (c) Within‐sample diversity measured through distance‐based Redundancy Analysis (dbRDA) plot. Shapes indicate the time of growth of plants whilst colours indicate their respective generation of growth. Data was analysed using permutational analysis considering the size effect and significance exerted by both the generations and time of growth. (d) Composition of bacterial taxa between the enrichment steps. The average relative abundance of taxa is shown in all generations (G0 to G4) and for all times of growth. The different colours of the bar charts indicate the time of growth of the plant. The standard deviation for each genus in samples is shown.

The average wet weight of roots and shoot aerial parts in 2‐week‐old plants showed a decline from G1 to G4 (Figure 2a). At G1, the roots weighed approximately 2.3 g and the shoots 2.8 g, compared to 1.7 g for both roots and shoots at G4 (Ordinary one‐way ANOVA, *p* < 0.0001). For plants grown for 4 weeks, root weight remained consistent across the four enrichment steps, whilst shoot wet weight decreased significantly, dropping from 5.45 g at the first enrichment step to 2.9 g at the fourth step (Ordinary one‐way ANOVA, *p* < 0.0001; Figure 2a).

**FIGURE 3:**
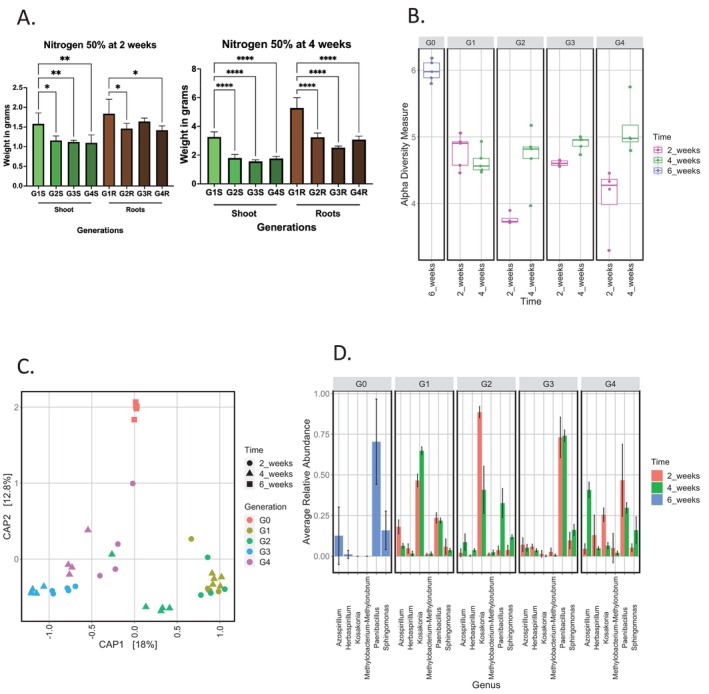
Plant growth parameters and rice root endobacteriome variations in the Nitrogen starved group at 50%. (a): Barcharts showing the variations of weight in grams of both roots and shoots of rice plants for the 4 generations of growth in Hoagland. The Graph on the left shows data of plants grown for 2 weeks at each generation, whilst the right‐side graph shows data of plants grown for 4 weeks at each generation. Statistical analysis was performed using ordinary one‐way ANOVA on Prism. Number of * indicate degree of significance. (**** indicate *p*‐value < 0.0001; whilst * indicates *p*‐value < 0.05). (b): Alpha diversity measure using Shannon throughout the generations at both 2 and 4 weeks of growth at each generation. Colours of the boxplots indicate a different time of growth. Differences are compared with Kruskal‐Wallis test and pairwise posthoc comparisons were calculated with Wilcoxon posthoc test. The *p*‐values corrections were made using Bonferroni correction. (c) Within sample diversity measured through distance‐based ReDundancy Analysis (dbRDA) plot. Shapes indicate the time of growth of plants whilst colours indicate their respective generation of growth. Data was analysed using permutational analysis considering the size effect and significance exerted by both the generations and time of growth. (d) Composition of bacterial taxa between the enrichment steps. The average relative abundance of taxa is shown in all generations (G0 to G4) and for all time of growth. The different colours of the barcharts indicate the time of growth of the plant. The Standard deviation for each genus in samples is shown.

For the nitrogen‐starved plants at 50%, the shoot mass of rice plants grown for 2 weeks significantly decreased from G1 (around 1.56 g) to G4 (around 1.06 g; Ordinary one‐way ANOVA; *p*‐values < 0.005) (Figure 3a). The same reduction was measured for the root biomass (1.87 g at G1 and 1.44 g at G4; Ordinary one‐way ANOVA; *p*‐values < 0.05). The shoot mass of plants grown for 4 weeks under nitrogen depletion also decreased from G1 (around 3.16 g) to G4 (around 1.83 g) (Figure 3a; Ordinary one‐way ANOVA *p*‐value < 0.001). The same reduction was detected for the root biomass (5.16 g at G1 and 3.16 g at G4; Ordinary one‐way ANOVA *p*‐value < 0.0001) with the average root mass that was higher than the average shoot mass. The same patterns of root and shoot weight reduction throughout the generations were noticed under N starved conditions at 75% both at 2 and 4 weeks of growth (Supporting Information; Figure 2a).

### 3.3 The Root Endophyte Bacteriome Undergoes Fluctuations and Changes in Composition Across Generations and Plant Growth Stages

It was of interest to understand, through 16S rRNA sequencing, how the endophytic community changes across different generations and to identify which community members are best suited to live in the rice endosphere. It was therefore hypothesized a decrease of species diversity (α‐ diversity) with an enrichment of specific endophytic bacteria at each re‐inoculation step.

The total number of reads, after filtering and removal of chloroplasts and mitochondria, ranged between 1136 and 56,206 with an average of 8110.92 per sample, spanning 9121 different ASVs. The α‐diversity, assessed by the Shannon index, ranged from 4.0 to 5.98 and showed a few significant variations across generations and plant stage development (i.e., 2 or 4 weeks of growth). Significant difference between G0 and all the other generations at 2 weeks of growth was observed (*p*‐value < 0.05), and between G0 and all generations of growth except G4 at 4 weeks of growth (*p*‐value < 0.05), whilst within the subsequent enrichment steps (G1 to G4), biodiversity stabilised with small and mostly non‐significant fluctuations (Figure 2b; Table S1). Importantly, the greatest difference observed between G0 and the other generations also coincides with the change in the growth medium used, which undoubtedly influenced the biodiversity composition amongst the communities.

The analysis of β‐diversity (Figure 2c) measured using a distance‐based redundancy analysis (dbRDA) on Bray–Curtis dissimilarity matrices, confirmed the results of the α‐diversity (Figure 2b). Importantly, we noticed that both the generation and the plant growth stage significantly influenced bacterial root endophytic community (ANOVA; *p*‐value < 0.001***). Rice samples from each generation formed distinct clusters along the first axis (14%) of the dbRDA plot, whilst plants grown for 2 weeks clustered separately from samples of rice grown for 4 weeks along the second axis (10.7%) of the plot (Figure 2c; Table S2). Interestingly, G3 and G4 at both time points were similar, suggesting a possible stabilisation of the endo‐microbiome at the third generation. This indicates that both the generation and growth duration significantly impact microbial diversity and community structure.

With the aim of identifying endophytes that best colonised rice roots, we compared the bacteriome present in each enrichment step via multivariate association using linear models' analysis. In rice plants grown for 2 weeks (Figure 2d), the taxonomic composition was mainly dominated by *Herbaspirillum, Kosakonia*, and *Methylobacterium‐Methylorubrum*, which were enriched in G1–G4 compared to roots of rice grown in soil (G0). Interestingly, *Paenibacillus* displayed an important reduction in abundance from G0 to G1 (71.5% to 4.1%; Figure S1) but exhibited a steady increase from G1 to G4, reaching 31.4%. Similarly, *Sphingomonas* declined from 18.9% at G0 to 1.9% at G1 but rose to 5.7% by G4, whilst *Azospirillum* followed a comparable trend but with fluctuations across generations (5.1% at G0 to 2.5% at G1 and then 6.4% at G2 and 3.1% at G3; Figure S1). These patterns suggest a bottleneck effect for these genera from G0 to G1, followed by successful re‐colonisation and persistence under the tested conditions in a context of low competition for colonisation and a low‐diversity microbiome. *Herbaspirillum* and *Methylobacterium‐Methylorubrum* showed fluctuations in abundance across G1 to G4 but remained consistently present. Interestingly, *Kosakonia*, undetectable in G0 (0% of abundance; Figure S1a), was significantly enriched in the further generations, especially at G1 (66.4%; Figure S1a) exceeding the abundances detected for all the other genera. The three genera which were enriched in plants at 2 weeks of growth, i.e., *Herbaspirillum, Methylobacterium‐Methylorubrum* and *Kosakonia*, were also enriched in 4 weeks old rice plants with some differences. *Kosakonia* was present at G1 in much lower abundance compared to the same generation at 2 weeks of growth (7.2% of abundance; Figure S1a) and was almost not detected in the other enrichment steps (0.1% of abundance at G2 and < 0.1% at G3 and G4; Figure S1a). The genera *Methylobacterium* and *Methylorubrum* were enriched and was not detected at G0, with fluctuating abundances in G1–G4, with the highest peak at G2 in 4 weeks old plants (8.3% of abundance; Figure S1a) (Figure 2d). At 4 weeks of growth, *Paenibacillus* and *Sphingomonas* followed similar trends as observed in 2‐week‐old plants, with initial reductions from G0 to G1, followed by increases to 34% and 11.7%, respectively, by G4. These observations highlight the dynamic shifts in microbial composition during the enrichment steps.

In summary, we observed a significant change in the structure and composition of the community driven by the enrichment steps, particularly between G0 and all subsequent generations. At the same time, we found that the plant growth duration (i.e., 2 or 4 weeks) greatly influenced the microbial community structure and is therefore an important parameter to consider when studying endophytic communities. Nevertheless, beyond these factors affecting the structure of the community, a clear set of endophytic bacteria were consistently enriched and maintained throughout the enrichment steps, regardless of the plant growth stage (2 or 4 weeks) and they represent a successful group of persistent rice endophytes.

### 3.4 The Nitrogen‐Depletion Condition Affects the Root Endophyte Bacteriome Across Generations and Plant Growth Stages

In order to investigate (i) how the endophytic bacterial community changed when the plant was subjected to an abiotic stress such as nitrogen starvation, (ii) how this community differed from plants grown under no stress, and (iii) to identify a set of super‐endophytes alleviating the abiotic stress, the same experimental procedure described above was performed under two N depletion conditions, i.e., at 50% and 75% of nitrogen depletion.

Under 50% nitrogen (N) depletion, there was a significant reduction in bacterial biodiversity measured using the Shannon index, in rice plants grown for 2 weeks (from 4.9 at G1 to 4.27 at G4), compared to those grown in soil at G0 without N depletion (around 5.9, *p*‐value < 0.5; Figure 3b). In between G1 and G4, the biodiversity values fluctuated but remained below those detected in G0. In the 4‐week N‐starved group (Figure 3b), there was a significant reduction in α‐diversity from G0 (around 5.9; *p*‐value < 0.05), although an increasing trend in diversity was observed across the enrichment steps, peaking at G4.

For plants grown for 2 weeks, the dbRDA plot (Figure 3c) showed distinct clustering of the root endophytic community from soil‐grown plants (G0) compared to all enrichment steps under 50% nitrogen depletion, with separation mainly along the first (18%) and second (12.8%) axes. Generations of enrichment clustered separately, though there was overlap between G1 and G2 (*p*‐value < 0.001). A similar clustering pattern was observed in plants grown for 4 weeks, where G0 samples clustered separately from those of the enrichment steps, primarily along the second axis, consistent with results from non‐stressed plants. This suggests that both generation and time of growth influenced bacterial community structure.

In line with all previous observations, under 75% nitrogen depletion, significant clustering based on the time of growth was observed along the second axis of the dbRDA plot (CAP2 13.5%; *p*‐value < 0.001). However, the most pronounced separation occurred between G0 and the subsequent generations, both at 2‐ and 4‐week growth stages, along the first axis (CAP1 16.3%) (Supporting Information; Figure 2c).

To identify which set of endophytes was enriched under nitrogen‐starved conditions, we conducted the same multivariate comparison analysis outlined above (Figure 3d). In rice plants grown under 50% nitrogen depletion for 2 weeks (G1‐G4) compared to those grown in soil (G0), the genera *Azospirillum, Herbaspirillum, Methylobacterium_Methylorubrum* and *Kosakonia* were significantly enriched as in the non‐stressed group. Their abundances fluctuated across enrichment steps without a consistent increasing pattern from G1 to G4. *Kosakonia* was highly abundant in G1 (30.71% of abundance; *Heatmap shown in supplementary material* Figure S1b) and G2 (39.04% of abundance; Figure S1b) but decreased in subsequent enrichment steps (8.74% of abundance in G4; Figure S1b). In 4‐week‐old rice plants under 50% nitrogen depletion, *Azospirillum, Herbaspirillum* and *Kosakonia* were enriched with a similar pattern (Figure 3d), but *Azospirillum* and *Herbaspirillum* were more abundant at 4 weeks of growth in G4 (25.81% and 3.06% respectively; Figure S1b) compared to 2‐week‐old rice plants (1.97% and 2.71% respectively; Figure S1b) whilst *Kosakonia* showed higher abundance in 2‐week‐old rice plants throughout the generations (i.e., 39.04% at 2 weeks compared to 24.59% at 4 weeks in G2; Figure S1b). Also, *Methylobacterium_Methylorubrum* was enriched in both 2‐ and 4‐week‐old rice plants compared to G0 in fluctuating abundances (i.e., from 0% at G0 to 0.88% in G1 at 2‐weeks and 1.48% in G2 at 4‐weeks; Figure S1b). Similar to the non‐stressed group, *Paenibacillus* exhibited a bottleneck effect, with its highest abundance in soil‐grown roots at G0 (78.29%; Figure S1b) and a significant reduction in subsequent generations. In generations grown in the artificial medium, it reached its peak abundance at G3 (31.64% and 45.02% in 2‐ and 4‐week‐old rice plants, respectively; Figure S1b).

For plants grown under 75% nitrogen depletion, abundance trends were similar to those observed in the 50% nitrogen‐depleted group. Notably, the genus *Ferrovibrio* was detected across all enrichment steps only in 4‐week‐old rice roots and showed higher enrichment at G3 compared to G0 (3.51% at G0 and 4.90% at G3; Figure S1b).

In summary, we found that even under abiotic stress caused by nitrogen deficiency, the endophytic community exhibits a response highly similar to the non‐stressed condition, characterised by a bottleneck effect from G0 to subsequent generations and notable variations in community structure and diversity across different plant growth stages. The strains identified as super‐endophytes, which are best adapted to persist in the endosphere under non‐stressed conditions, remained largely unchanged under nitrogen stress. However, *Ferrovibrio* showed a significant increase in abundance under abiotic stress compared to the non‐stressed condition.

## 4 Discussion

Scientists have recently been interested in isolating endophytes from many different plants, and several microbiome studies have also highlighted the diversity of microbes colonising the endosphere. There are methodological constraints and a lack of standardisation in the protocols for their isolation, as well as for DNA purification for culture‐independent studies of endosphere microbial communities (Compant et al. 2021). Consequently, culture‐independent studies show variability in microbial communities, and culture‐dependent studies result in the isolation of not true endophytes since, in most cases, *in planta* experiments are not performed as a follow‐up of endophyte isolation. Here, we combined laborious culture‐dependent and culture‐independent techniques to stringently enrich for endophytes. The *in planta* enrichment methods used here can prove useful in the targeted isolation of endophytes and their application to increase plant health.

In our approach, we observed a constant weight reduction from G1 to G4 of both roots and shoot aerial parts in both the non‐stressed plants. The reason for this in non‐stressed plants is currently unknown; however, as plants were grown in controlled conditions (i.e., Hoagland solution), this may have led to nutrient depletion and subsequently impacted plant growth. Bacterial populations in the root zone compete for essential nutrients, such as nitrogen, phosphorus, and trace elements, potentially limiting plant growth.

We expected that there would be a reduction of bacterial diversity from G0 to G4 followed by enrichment of specific bacterial genera. We observed a reduction of α‐diversity, from G0 to all stages going through G1‐G4. In G0, rice roots drove the recruitment of the endophytic microbiome from the soil, a primary source of microbial diversity (Hardoim 2015). The reduction of diversity from G0 to G1 was due to a selective pressure by the plant and to random variations of the abundances of the bacterial taxa. A study by (Jiao et al. 2019) showed that rare taxa are affected by abiotic disturbances in microbiomes probably due to their small population size. Each enrichment step can be considered as a unique event in which the root endophytic microbiome is influenced by a series of factors like root exudates (Edwards et al. 2015). The endophytic diversity from G1‐G4 at both time points did not vary significantly. This enrichment procedure resulted in bacteriome variation, since each generation, depending on the growth time, clustered separately. Hence, the main driver of variation was the type of growth substrate, the reinoculation of endophytic mixture and the growth time.

A few enrichment type studies on endophytes have been reported. For example, enriching root endophytes through isolation of specific strains using selective media or advanced methods such as flow cytometry (Carper et al. 2021). Endophytes were also enriched via a differential and density gradient centrifugation to separate endophytic bacteria from *Populus* roots to reduce contaminant DNA and to optimise bioinformatic analyses after single‐cell genomics (Utturkar et al. 2016). Another study showed the efficacy of atomizing a *Paraburkholderia* endophyte through the flowers of the parent plants to enrich the seed endophytic community in the offspring enrichment steps (Mitter et al. 2017). Jha et al. examined the effect on the rice endophytic community in plants treated with urea fertilisers and/or with a biofertilizer containing *Rhizobium leguminosarum bv. trifolii* E11 (Jha et al. 2020). Other studies have highlighted the importance of agricultural practises such as crop rotation and compost usage to maintain a high soil microbial diversity, resulting in a highly diverse and rich endophytic community (Araujo 2022). The approach here was to enrich and study the endophytic community using serial microbial transplantations/inoculations throughout generations of plants.

This *in planta* enrichment procedure identified ‘super’ endophytic bacteria which could successfully serially colonise and persist in rice roots. For example, it was evident that *Paenibacillus* was enriched with an increasing trend within the enrichment steps; *Paenibacillus* is a free‐living nitrogen fixing bacterium with plant growth promoting properties (Brito et al. 2020; Dixit et al. 2022; Liu et al. 2019). The genus was shown to be part of the rice core endophytic microbiome propagating from rice seeds to seedlings grown in a sterile environment, in line with our findings (Wang et al. 2020). Moreover, this genus was shown to have successful root colonisation and persistence abilities in other plant species (Garcia‐Lemos et al. 2020). A study has shown the role of *Paenibacillus peoriae* SP9 as efficient biocontrol agent against *Pythium* infestation and supporting plant growth in wheat roots rhizosphere and endosphere (Araujo et al. 2020). Interestingly, the study showed also fluctuating abundances of *P. peoriae* SP9 across plant growth stages as encountered in the present study. The genus *Kosakonia* was also enriched with fluctuating abundances. Strains of the diazotrophic genus *Kosakonia* were found to efficiently colonise rice roots and are recently receiving attention for their PGP properties (Berger et al. 2018; Li et al. 2017; Meng et al. 2015; Mosquito et al. 2020; Walitang et al. 2017). In fact, species pertaining to *Kosakonia* were found to produce Indole‐3‐Acetic Acid that stimulates root elongation and overall plant growth, to enhance plant nutrient availability through phosphate solubilisation and siderophore production, and to improve plant resistance and growth under abiotic stresses such as drought and salinity (Romano et al. 2020; Walitang et al. 2017). *Kosakonia* was found in extremely low abundance at G0 but increased significantly in G1 and G2. However, it was detectable in G3 and G4 only in plants grown for 2 weeks. Similarly, a field study reported that a *Kosakonia* strain inoculated on rice seeds was detectable only during the first weeks of growth, with its presence dropping sharply after 2 months (Mosquito et al. 2020). These findings suggest that *Kosakonia* acts as an early coloniser of rice roots, with its abundance diminishing in later growth stages. Moreover, its richness under nitrogen starved conditions could be linked to the presence of *nif* and *anf* gene clusters in *Kosakonia* genomes that enables the genus to fix nitrogen, providing an alternative N source in nitrogen limiting conditions (Quintas‐Nunes et al. 2022; Taulé et al. 2019). Similarly, the genus *Methylobacterium_Methylorubrum* was enriched at 2 and 4 weeks of growth in all groups. *Methylobacterium* is a nitrogen fixing plant growth promoting bacterium commonly found in rice seeds and in different plant tissues, establishing a stable endophytic relationship with plants, and is known to produce different phytohormones and to mobilise nutrients, promoting plant growth (Chaudhry et al. 2016; Jourand et al. 2004; Kutschera 2007; Madhaiyan et al. 2015; Tani et al. 2015; Walitang et al. 2017). *Methylobacterium* species were found to mitigate stress by reducing ethylene levels (Grossi et al. 2020). As for the genus *Paenibacillus*, *Methylobacterium* was found to be part of the core rice endophytic microbiome propagating in sterile environments and, in some plants, it constitutes a significant portion of the culturable endophytic community (Roodi et al. 2020; Wang et al. 2020). Interestingly, it was found that the presence of *Methylobacterium* species in rice leaves constitutes a health signature, reducing the severity of *Xanthomonas oryzae* infections by 77% (Lai et al. 2020; Oeum et al. 2024). These literature findings indicate a positive correlation between the rice plant and this genus, supporting our hypothesis of an enrichment of super endophytes within rice roots throughout the enrichment steps. We also evidenced that with nitrogen depleted at 50%, the genus *Ferrovibrio* displayed low abundance at the early stages (G0) whereas its abundance rose across generations in 4 weeks old rice plants. This genus has been commonly isolated as a root endophyte and it was shown to improve water quality by converting bromate, a polluting element, to bromide (C. Liu et al. 2020; Yang et al. 2022). Importantly, we observed that *Ferrovibrio'*s abundance increased only under nitrogen depletion. This fits into the ‘insurance’ hypothesis (Gonzalez et al. 2009; Matias et al. 2013; Yachi and Loreau 1999), which suggests that rare species in a microbial community can increase and contribute to ecosystem stability under changing environmental states. This concept is closely linked to functional redundancy, as low‐abundant taxa can increase in specific environmental conditions that favour their proliferation (Kurm et al. 2019), ensuring the maintenance of key ecosystem functions despite shifts in community composition. Another plausible hypothesis for its enrichment under N‐limiting conditions is the indirect effect induced by the plant microbiome modification that attracts bacteria able to fix nitrogen or involved in other nitrogen related processes. An example of this theory is detailed in *Stevia rebaudiana* , in which nitrogen deficiency modulated the abundance of rhizobacterial taxa under drought stress (Sun et al. 2024). However, an overall knowledge‐gap exists concerning the specific role and importance of this genus within the plant tissues. In most of the treated plant groups there was an enrichment of *Azospirillum*, a free‐living nitrogen fixing, and a common plant growth promoting rhizobacterium (Bao et al. 2013; Galindo et al. 2016; Raglin and Kent 2025; Yasuda et al. 2009). This genus was found to promote growth and nitrogen use efficiency of *Solanum tuberosum* L. (Naqqash et al. 2022). Finally, *Herbaspirillum* was enriched in all of the nitrogen starved groups; it is a free living diazotrophic genus comprising of many plant growth promoting species commonly found in association with rice in stems and root endosphere and rhizosphere (Alves et al. 2015; Chubatsu et al. 2012; Elbeltagy et al. 2001; Mano et al. 2006). *Herbaspirillum* sp. B501 colonises different rice internal tissues, establishing stable endophytic relationships and was found to fix nitrogen in rice plants providing an exogenous source of this element (Elbeltagy et al. 2001). All this data highlight that the methodology used here enriches bacterial endosphere communities, hence provides valuable information for the tailored isolation of endophytes under specific environmental conditions as well as for the design of microbial consortia.

To conclude, in this study we designed and performed a high throughput enrichment strategy to map and isolate rice root endosphere bacteria. In the future, experiments using a wider range of plants and a further trial on plants grown in bulk soil will ensure a more pertinent condition. To investigate the adaptability of endophytes under various environmental conditions, a further application of the technique will be performed on other abiotic stresses, of which increased salinity at different levels, and on other plant species. The initial study will be followed by a second applicable one, isolating and characterising strains enriched in this future analysis and testing them as inoculants as single or in consortia. If proven efficient, inoculants isolated through this enrichment strategy could find future prospects as biofertilizers and even biocontrol agents. The global shift towards eco‐friendly agricultural practises is driving an increase in the market for biofertilizers and biopesticides, which is projected to continue growing as farmers seek alternatives to harmful chemicals. The application of this technique using enriched endophytes as biofertilizers and biocontrol agents presents a promising frontier in sustainable agriculture. If selectively enriched for PGP traits, these microorganisms can serve as alternatives thereby lowering the reliance on chemical pesticides. This contributes to soil health and biodiversity, responding to growing consumer demands for organic produce and improving market viability for products cultivated with these natural methods. Utilising endophytes enhances promotes a circular economy through the recycling of organic matter. Additionally, these practises resonate with societal values centred around environmental sustainability, reducing chemical residues in food supplies, and preserving biodiversity. As such, the integration of enriched super‐endophytes into agricultural practises can create a pathway towards more sustainable food systems whilst catering to economic viability and public health concerns.

## Author Contributions

D.K.C. conducted the study and was actively involved in the experimental design, data interpretation, and manuscript writing. I.B. provided overall supervision of the research. A.E. and S.P. contributed to the analysis of the microbiome sequencing data. C.B. and V.V. conceptualized and designed the experiments, supervised the project, contributed to data interpretation, and were involved in drafting and revising the manuscript.

## Ethics Statement

The authors have nothing to report.

## Consent

The authors have nothing to report.

## Conflicts of Interest

The authors declare no conflicts of interest.

## Supporting information


**Table S1.** Differences in alpha diversity. Generations and Time were compared using Kruskal test. Pairwise posthoc comparisons were calculated using Wilcoxon posthoc test. The *p*‐values were corrected for multiple comparisons using Bonferroni correction. ST1A shows statistical data related to the non‐stressed group at 2 and 4 weeks of growth for Generations 0, G1, G2, G3 and G4. ST1B shows statistical data related to the Nitrogen starved group at 50% at 2 and 4 weeks of growth for G0, G1, G2, G3 and G4. ST1C shows statistical data related to the Nitrogen starved group at 75% at 2 and 4 weeks of growth for G0, G1, G2, G3 and G4.
**Table S2.** Statistical analyses of the variations shown in the dbRDA plots. Permutational analysis was used to obtain size effects and significances for compositional differences of the bacterial community between Generation (i) and Time (ii). STA shows statistical data relative to the non‐stressed group. STB shows statistical data relative to the Nitrogen starved group at 50%. STC shows statistical data relative to the Nitrogen starved group at 75%.
**Figure S1.** Heatmap of Genus abundance by generation and time. The given percentage of abundance shown is relative only to the analysed strains and is not an absolute value. (A) shows the heatmap of enriched genus in the non stressed group. (B) Shows the heatmap of enriched genus in the nitrogen starved group at 50%. (C) Shows the heatmap of enriched genus in the nitrogen starved group at 75%.
**Figure S2.** Plant growth parameters and rice root endobacteriome variations in the Nitrogen starved group at 75%. (A) Barcharts showing the variations of weight in grams of both roots and shoots of rice plants for the 4 generations of growth in Hoagland. The Graph on the left shows data of plants grown for 2 weeks at each generation, whilst the right‐side graph shows data of plants grown for 4 weeks at each generation. Statistical analysis was performed using ordinary one‐way ANOVA on Prism. Number of * indicate degree of significance (**** indicate *p*‐value < 0.0001; whilst * indicates *p*‐value < 0.05). (B) Alpha diversity measure using Shannon throughout the generations at both 2 and 4 weeks of growth at each generation. Colours of the boxplots indicate a different time of growth. Differences are compared with Kruskal‐Wallis test and pairwise posthoc comparisons were calculated with Wilcoxon posthoc test. The *p*‐values corrections were made using Bonferroni correction. (C) Within sample diversity measured through distance‐based ReDundancy Analysis (dbRDA) plot. Shapes indicate the time of growth of plants whilst colours indicate their respective generation of growth. (D) Composition of bacterial taxa between the enrichment steps. The average relative abundance of taxa is shown in all generations (G0 to G4) and for all time of growth. The different colours of the barcharts indicate the time of growth of the plant. The Standard deviation for each genus in samples is shown.

## Data Availability

The data that support the findings of this study are available on request from the corresponding author. The data are not publicly available due to privacy or ethical restrictions.
