# Supplementary material for: An in Planta Enrichment Route to Identify Bacterial Root Endophytes
Source: Environ Microbiol Rep. 2025 Jun 19;17(3):e70136. doi: 10.1111/1758-2229.70136 (PMC12178614; doi:10.1111/1758-2229.70136)
Supplement: Supplementary file 1 — Table S1. Differences in alpha diversity. Generations and Time were compared using Kruskal test. Pairwise posthoc comparisons were calculated using Wilcoxon posthoc test. The p‐values were corrected for multiple comparisons using Bonferroni correction. ST1A shows statistical data related to the non‐stressed group at 2 and 4 weeks of growth for Generations 0, G1, G2, G3 and G4. ST1B shows statistical data related to the Nitrogen starved group at 50% at 2 and 4 weeks of growth for G0, G1, G2, G3 and G4. ST1C shows statistical data related to the Nitrogen starved group at 75% at 2 and 4 weeks of growth for G0, G1, G2, G3 and G4. Table S2. Statistical analyses of the variations shown in the dbRDA plots. Permutational analysis was used to obtain size effects and significances for compositional differences of the bacterial community between Generation (i) and Time (ii). STA shows statistical data relative to the non‐stressed group. STB shows statistical data relative to the Nitrogen starved group at 50%. STC shows statistical data relative to the Nitrogen starved group at 75%. Figure S1. Heatmap of Genus abundance by generation and time. The given percentage of abundance shown is relative only to the analysed strains and is not an absolute value. (A) shows the heatmap of enriched genus in the non stressed group. (B) Shows the heatmap of enriched genus in the nitrogen starved group at 50%. (C) Shows the heatmap of enriched genus in the nitrogen starved group at 75%. Figure S2. Plant growth parameters and rice root endobacteriome variations in the Nitrogen starved group at 75%. (A) Barcharts showing the variations of weight in grams of both roots and shoots of rice plants for the 4 generations of growth in Hoagland. The Graph on the left shows data of plants grown for 2 weeks at each generation, whilst the right‐side graph shows data of plants grown for 4 weeks at each generation. Statistical analysis was performed using ordinary one‐way ANOVA on Prism. Number of * indicate [file EMI4-17-e70136-s001.docx]

SUPPLEMENTARY MATERIAL


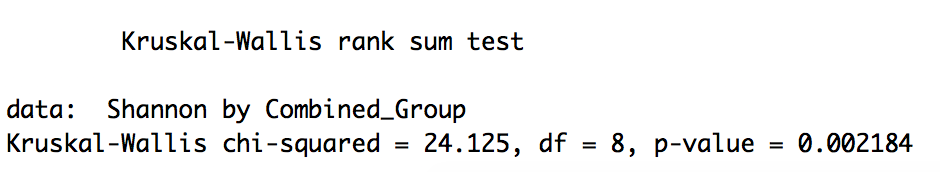

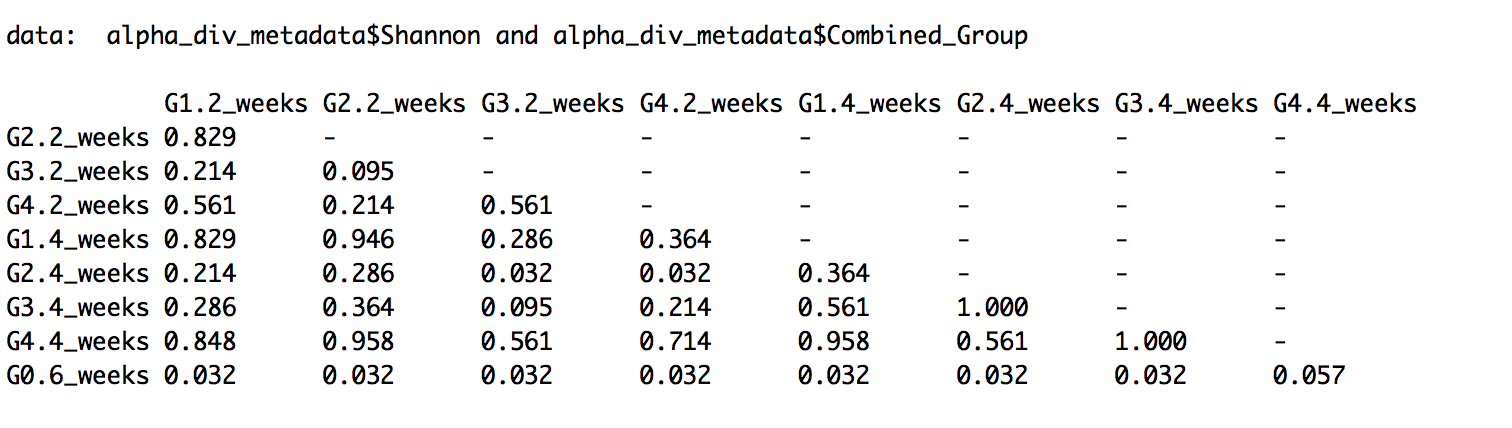


A.


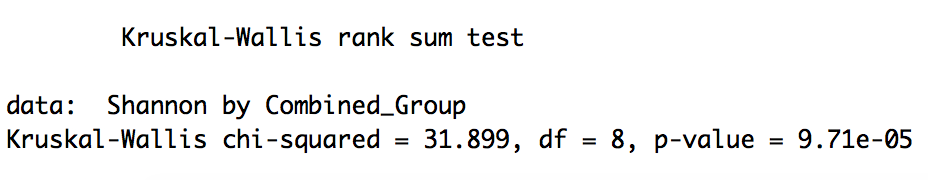

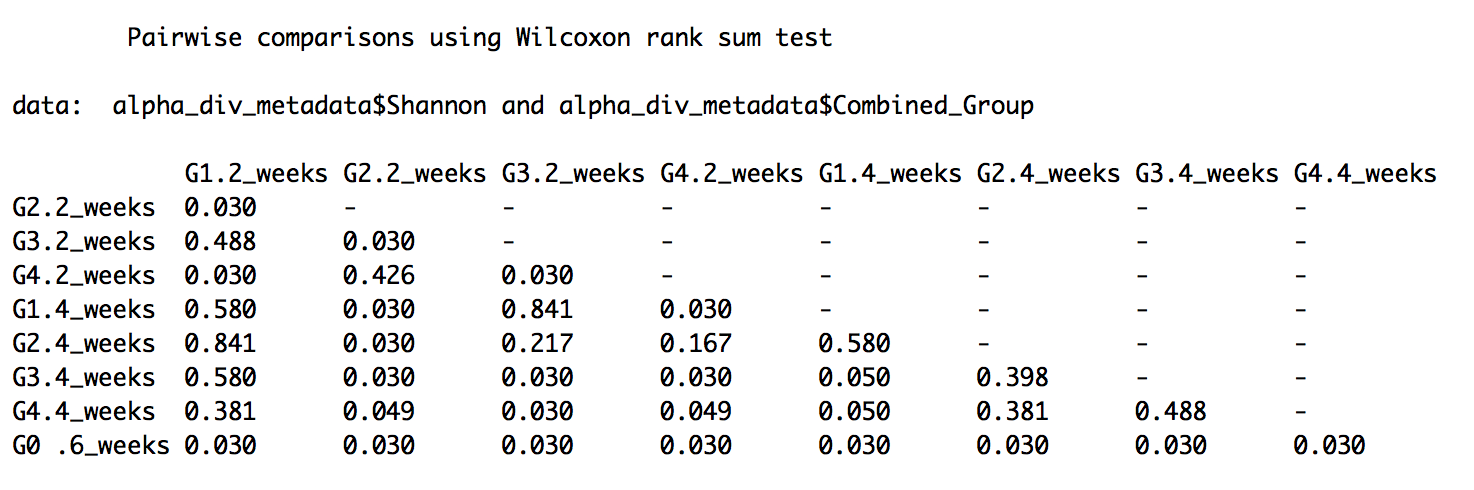


B.


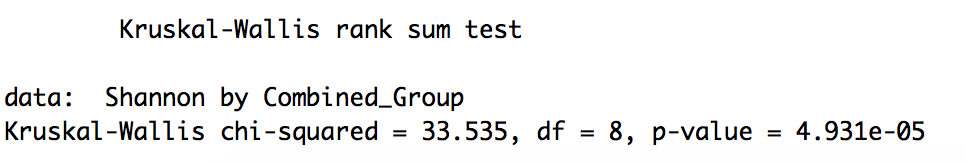

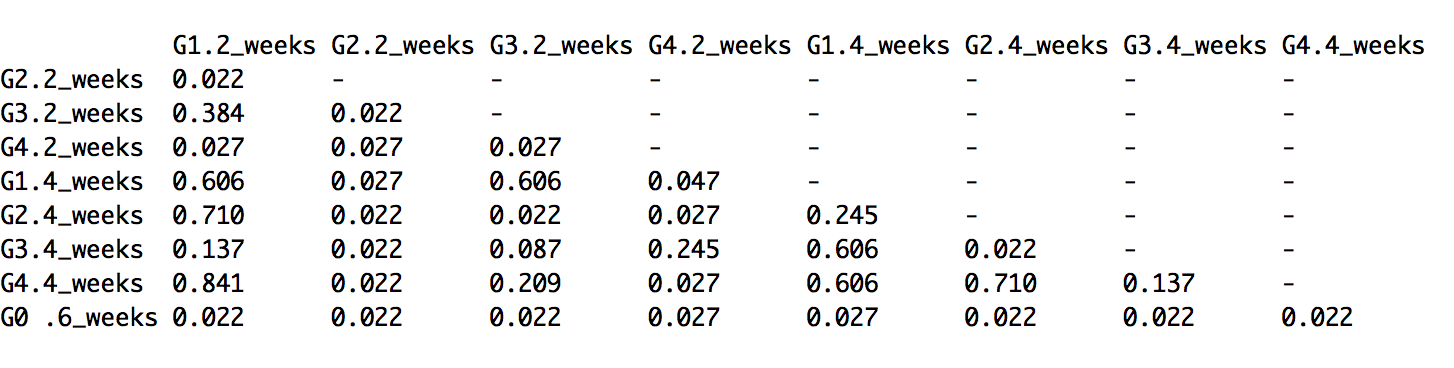


C.

Supplementary Table 1. Differences in alpha diversity. Generations and Time were compared using Kruskal test. Pairwise posthoc comparisons were calculated using Wilcoxon posthoc test. The p-values were corrected for multiple comparisons using Bonferroni correction.

ST1A shows statistical data related to the non stressed group at 2 and 4 weeks of growth for Generations 0, G1, G2, G3 and G4.

ST1B shows statistical data related to the Nitrogen starved group at 50% at 2 and 4 weeks of growth for G0, G1, G2, G3 and G4.

ST1C shows statistical data related to the Nitrogen starved group at 75% at 2 and 4 weeks of growth for G0, G1, G2, G3 and G4.


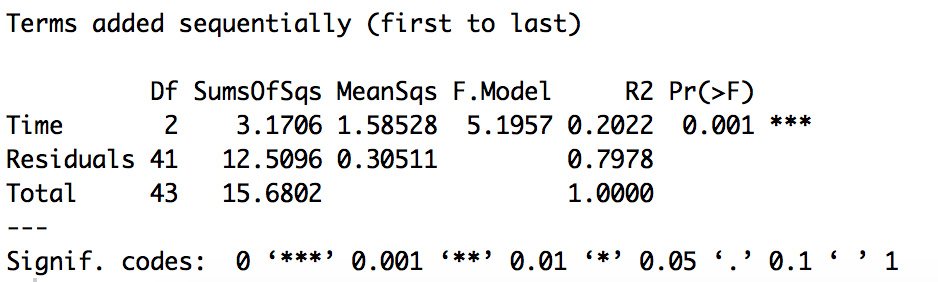

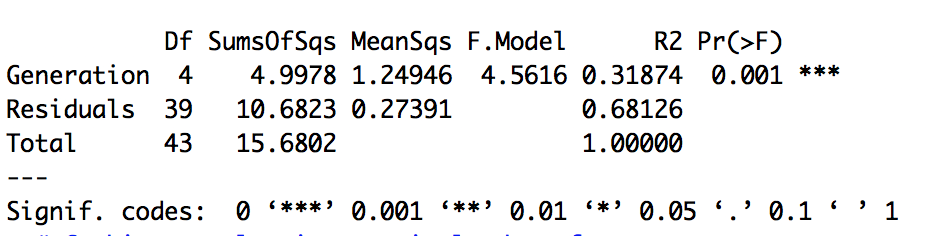


A.

B.


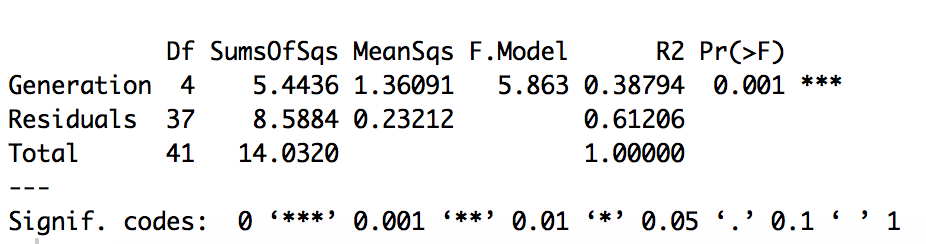

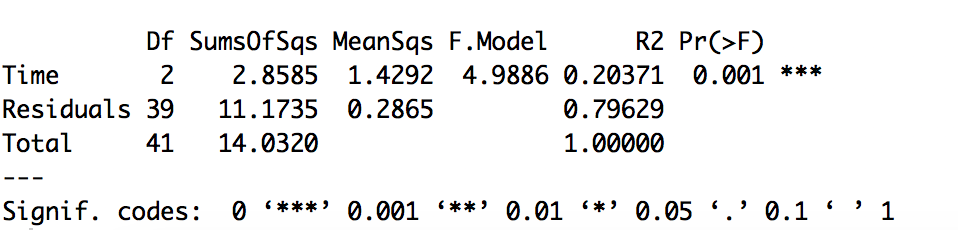


i.

ii.

i.

ii.


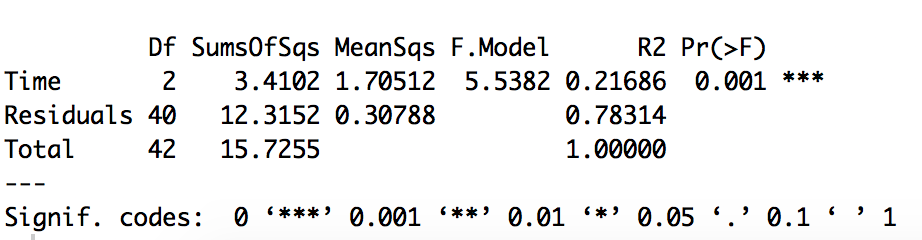

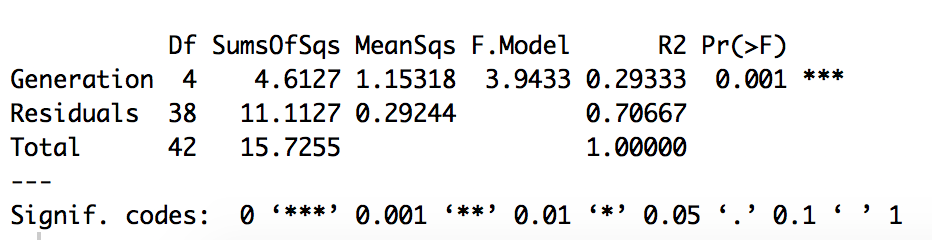


C.

i.

ii.

Supplementary Table 2. Statistical analyses of the variations shown in the dbRDA plots. Permutational analysis was used to obtain size effects and significances for compositional differences of the bacterial community between Generation (i) and Time (ii)

STA shows statistical data relative to the non stressed group.

STB shows statistical data relative to the Nitrogen starved group at 50%.

STC shows statistical data relative to the Nitrogen starved group at 75%.


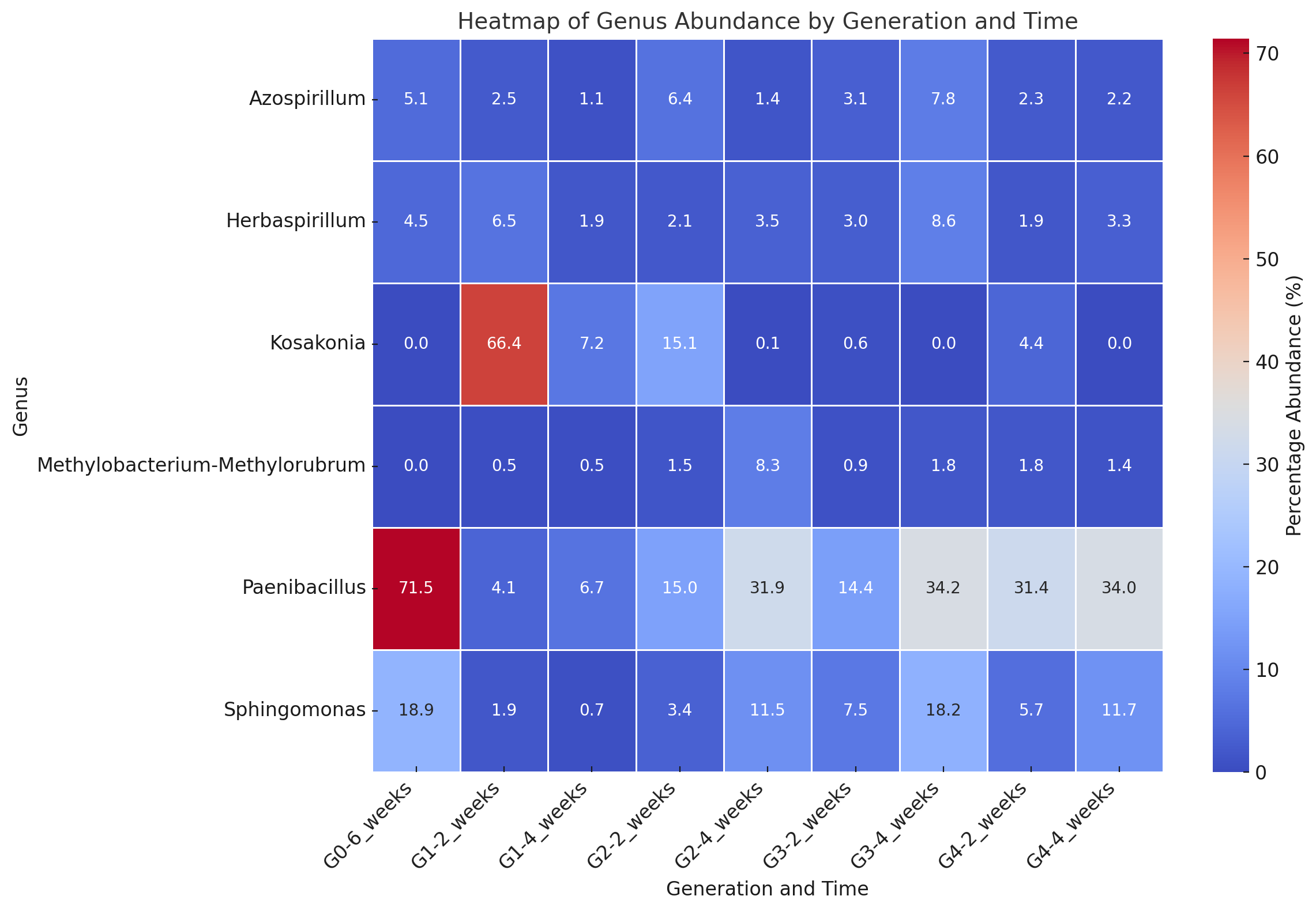

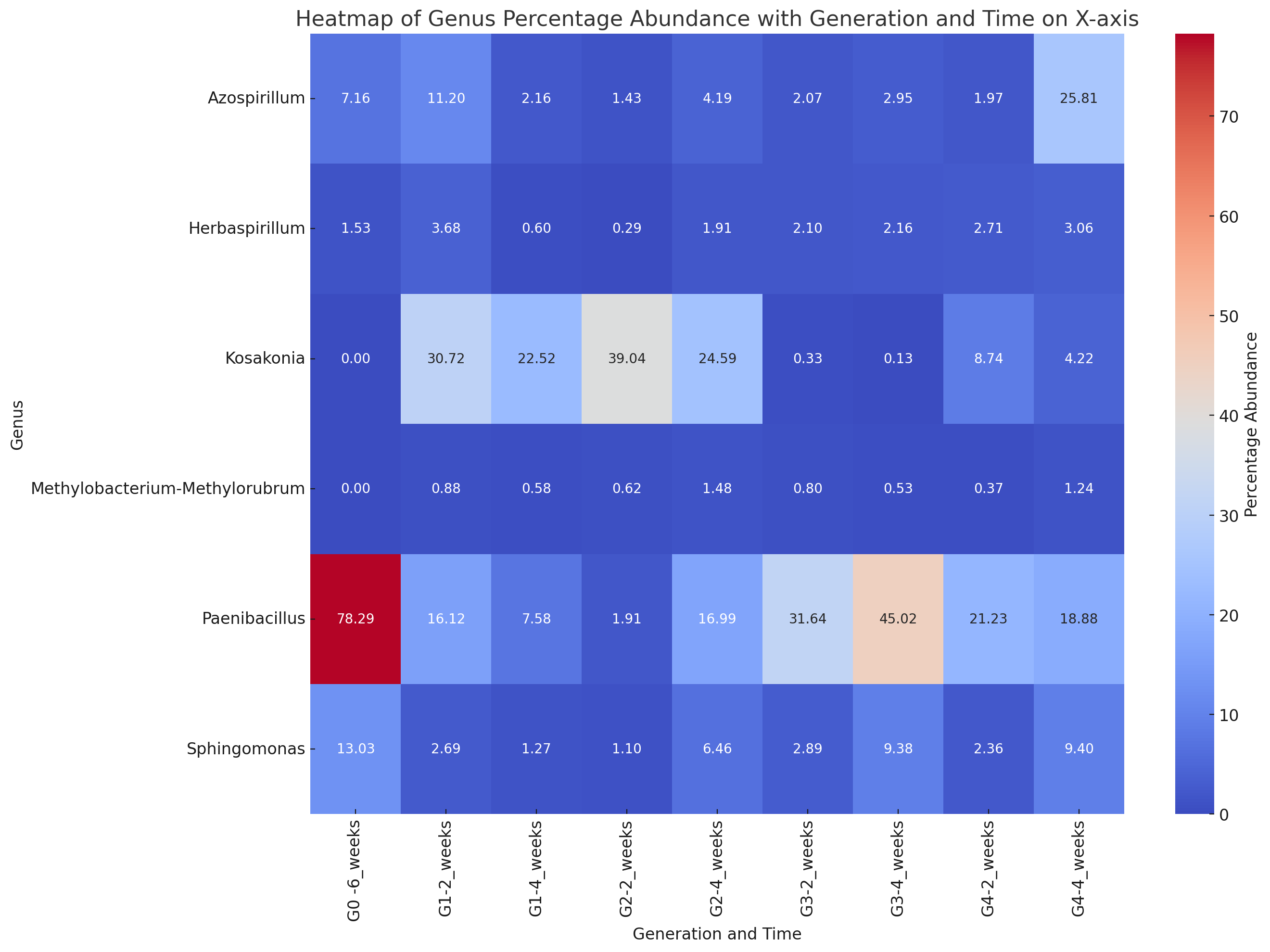


A.

B.


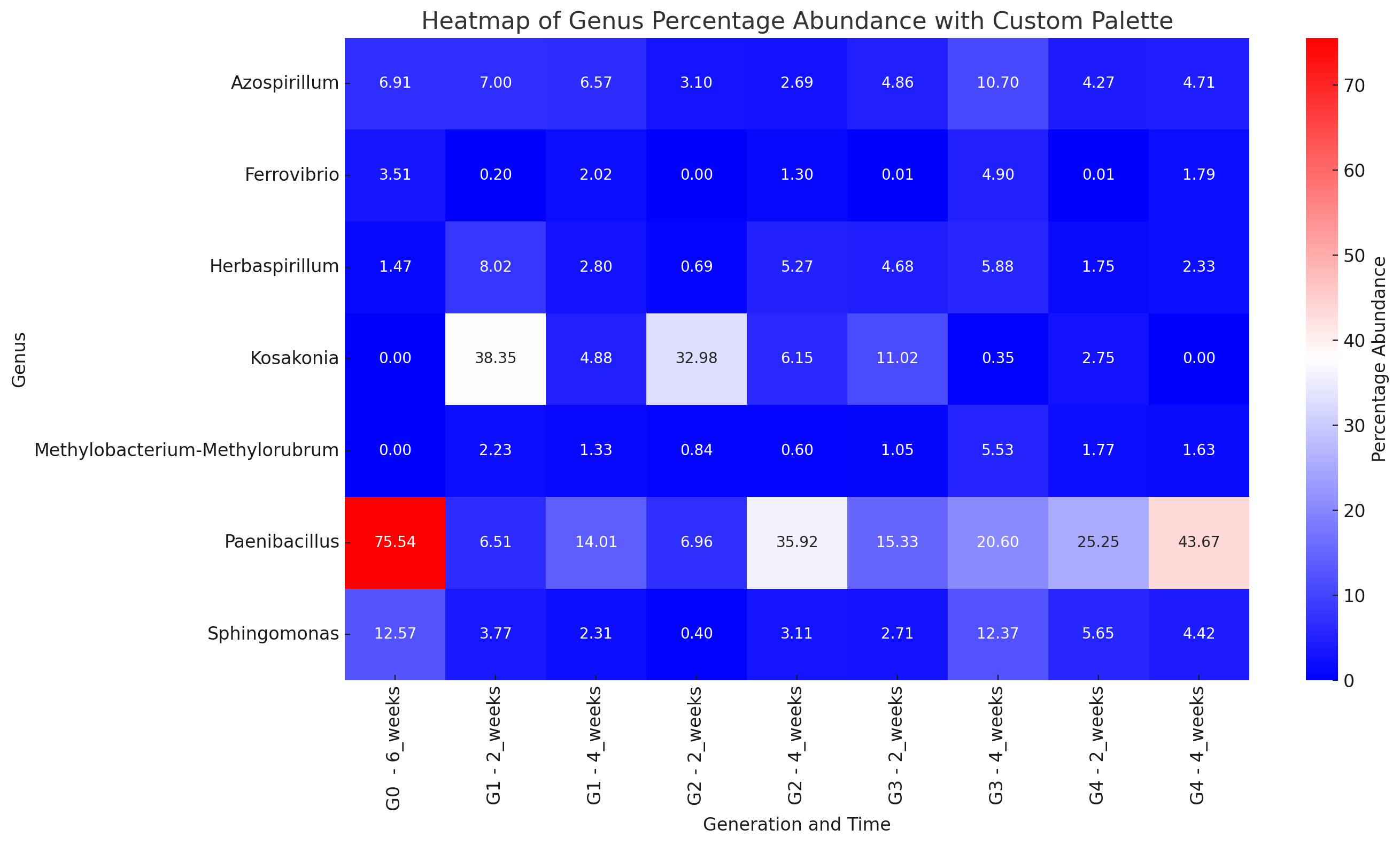


C.

Supplementary figure 1. Heatmap of Genus abundance by generation and time. The given percentage of abundance shown is relative only to the analyzed strains and is not an absolute value.

Figure S1A shows the heatmap of enriched genus in the non stressed group.

Figure S1B shows the heatmap of enriched genus in the nitrogen starved group at 50%.

Figure S1C shows the heatmap of enriched genus in the nitrogen starved group at 75%.

A.

B.

C.

D.


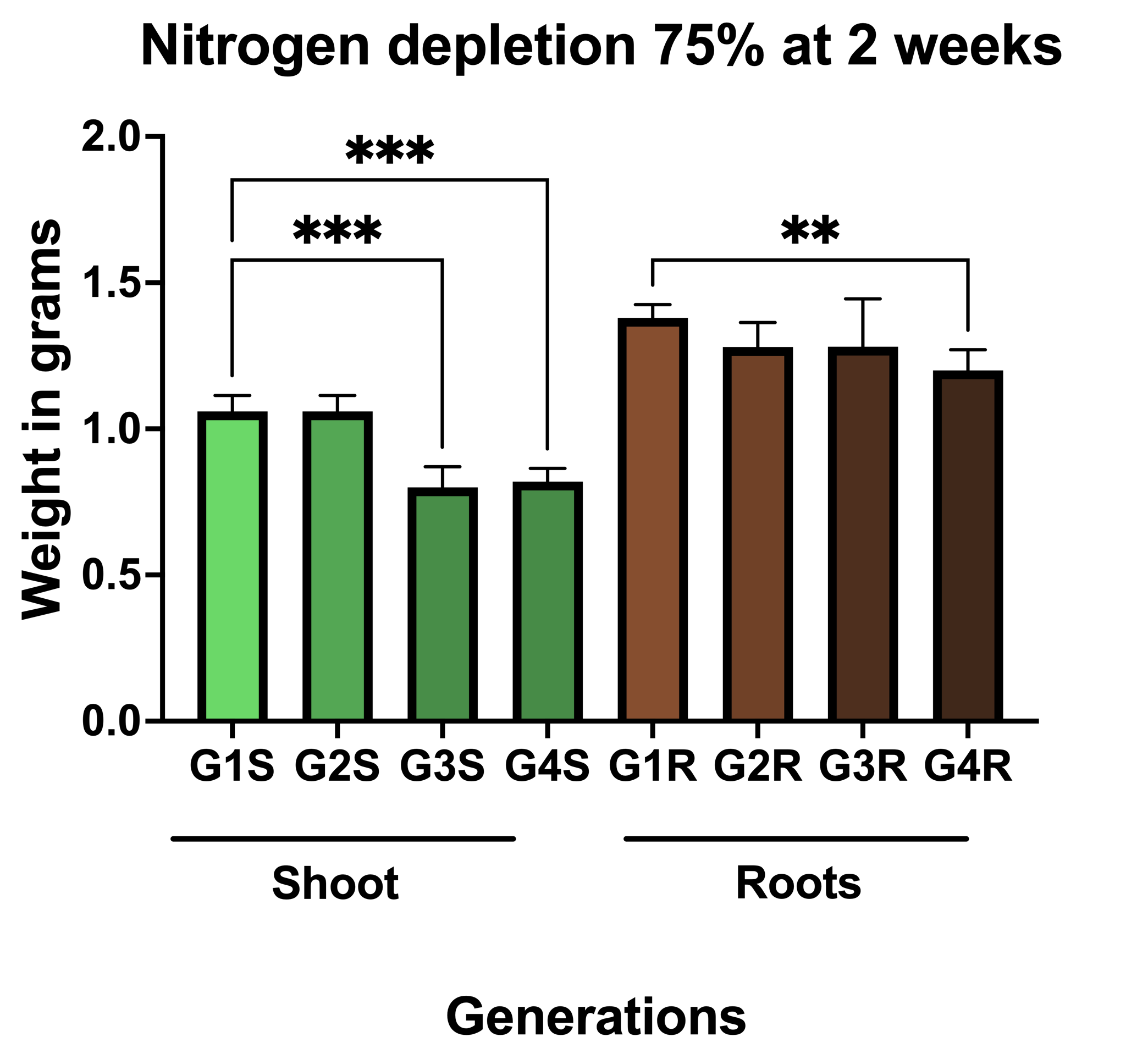

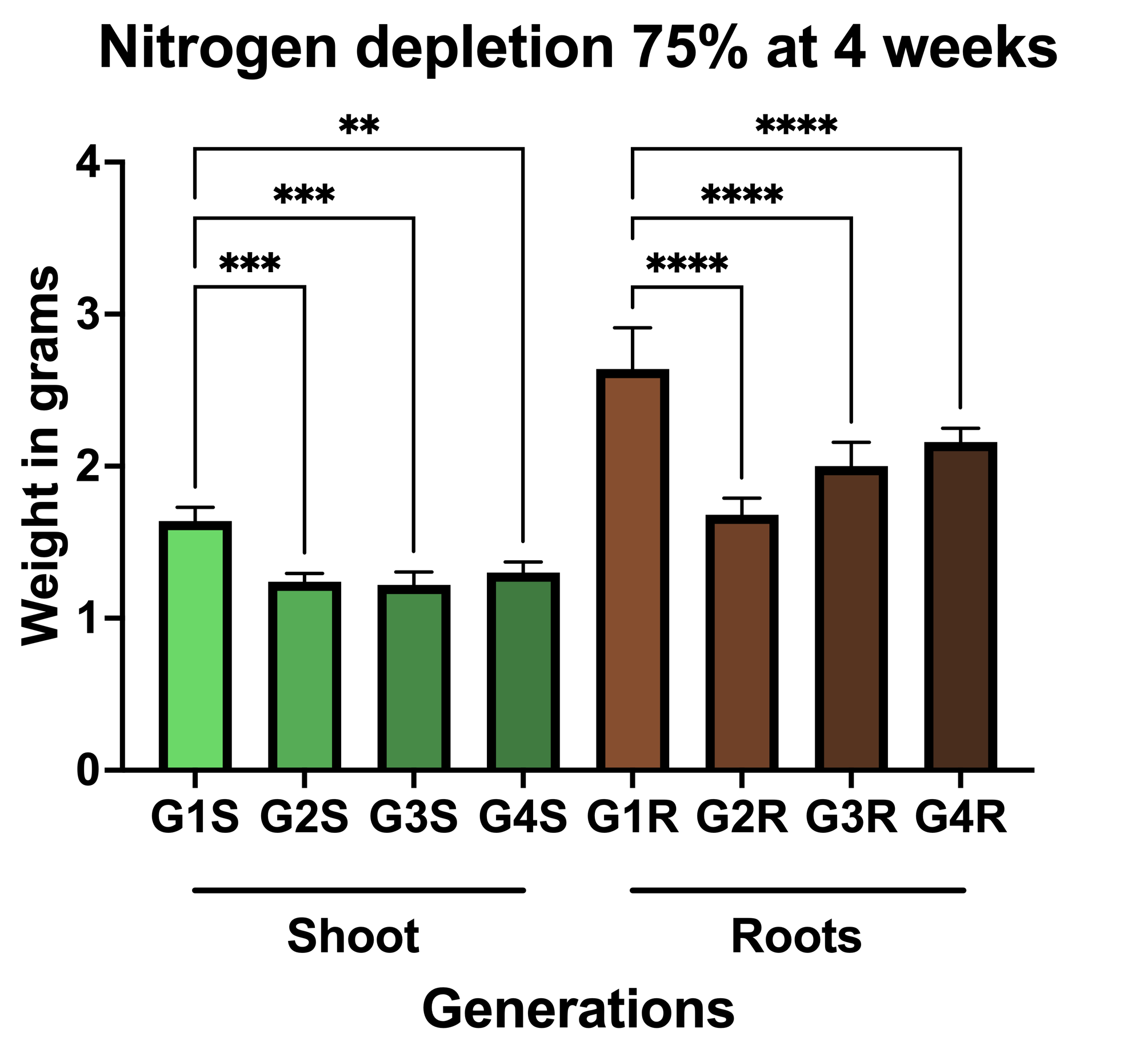


Supplementary figure 2. Plant growth parameters and rice root endobacteriome variations in the Nitrogen starved group at 75%.

Fig2A: Barcharts showing the variations of weight in grams of both roots and shoots of rice plants for the 4 generations of growth in Hoagland. The Graph on the left shows data of plants grown for 2 weeks at each generation, while the right-side graphs shows data of plants grown for 4 weeks at each generation. Statistical analysis was performed using ordinary one-way ANOVA on Prism. Number of * indicate degree of significance. (**** indicate p-value < 0.0001; while * indicates p-value < 0.05).

Fig2B: Alpha diversity measure using Shannon throughout the generations at both 2 and 4 weeks of growth at each generation. Colors of the boxplots indicate a different time of growth. Differences are compared with Kruskal-Wallis test and pairwise posthoc comparisons were calculated with Wilcoxon posthoc test. The p-values corrections were made using Bonferroni correction.

Fig2C: Within sample diversity measured through distance-based ReDundancy Analysis (dbRDA) plot. Shapes indicate the time of growth of plants while colors indicate their respective generation of growth.

Fig2D: Composition of bacterial taxa between the enrichment steps. The average relative abundance of taxa is shown in all generations (G0 to G4) and for all time of growth. The different colors of the barcharts indicate the time of growth of the plant. The Standard deviation for each genus in samples is shown.
